# Human electronegative low-density lipoprotein modulates cardiac repolarization via LOX-1-mediated alteration of sarcolemmal ion channels

**DOI:** 10.1038/s41598-017-10503-x

**Published:** 2017-09-07

**Authors:** An-Sheng Lee, Yutao Xi, Chin-Hu Lai, Wei-Yu Chen, Hsien-Yu Peng, Hua-Chen Chan, Chu-Huang Chen, Kuan-Cheng Chang

**Affiliations:** 1 Department of Medicine, Mackay Medical College, New Taipei, Taiwan; 2 Cardiovascular Research Laboratory, China Medical University Hospital, Taichung, Taiwan; 3 Texas Heart Institute/St. Luke’s Hospital, Houston, TX USA; 4 Graduate Institute of Biomedical Sciences, China Medical University, Taichung, Taiwan; 5 Department of Surgery, Taichung Armed Forces General Hospital, Taichung, Taiwan; 6 Center for Lipid Biosciences, Kaohsiung Medical University Hospital, Kaohsiung, Taiwan; 7 Vascular and Medicinal Research, Texas Heart Institute, Houston, TX USA; 8 Division of Cardiovascular Medicine, China Medical University Hospital, Taichung, Taiwan; 9 Graduate Institute of Medicine, College of Medicine, Kaohsiung Medical University, Kaohsiung, Taiwan; 10 Lipid Science and Aging Research Center, Kaohsiung Medical University, Kaohsiung, Taiwan

## Abstract

Dyslipidemia is associated with greater risk of ventricular tachyarrhythmias in patients with cardiovascular diseases. We aimed to examine whether the most electronegative subfraction of low-density lipoprotein (LDL), L5, is correlated with QTc prolongation in patients with coronary artery disease (CAD) and investigate the effects of human L5 on the electrophysiological properties of cardiomyocytes in relation to the lectin-like oxidized LDL receptor (LOX-1). L5 was isolated from the plasma of 40 patients with angiography documented CAD and 13 patients with no CAD to correlate the QTc interval respectively. The mean concentration of L5 was higher and correlated with QTc in patients with CAD compared to controls. To examine the direct effect of L5 on QTc, mice were intravenously injected with L5 or L1. L5-injected wild-type but not LOX-1^−/−^ mice showed longer QTc compared to L1-injected animals *in vivo* with corresponding longer action potential duration (APD) in cardiomyocytes incubated with L5 *in vitro*. The APD prolongation was mediated by an increase of L-type calcium current and a decrease of transient outward potassium current. We show that L5 was positively correlated with QTc prolongation in patients with ischemic heart disease. L5 can modulate cardiac repolarization via LOX-1-mediated alteration sarcolemmal ionic currents.

## Introduction

Dyslipidemia, characterized by high plasma levels of low-density lipoprotein (LDL) cholesterol and low levels of high-density lipoprotein (HDL) cholesterol is an independent risk factor for coronary artery disease and myocardial infarction^1–4^. Numerous randomized controlled trials have demonstrated a robust beneficial effect by LDL-lowering therapy to reduce major adverse cardiovascular events including myocardial infarction, stroke and all-cause mortality in primary or secondary prevention settings^5–10^. Since about half of all deaths due to cardiovascular diseases may manifest as ventricular tachycardia/fibrillation (VT/VF) related sudden cardiac death, it raised an important question that some of the beneficial effects of lipid-lowering therapy may be attributed to the reduction of ventricular tachyarrhythmias and sudden death. Indeed, a number of previous studies have demonstrated that lipid-lowering therapy with statins was associated with a lower risk of developing VT/VF in patients with coronary artery disease or nonischemic cardiomyopathy^11–15^. Interestingly, a recent meta-analysis showed that reducing LDL cholesterol with a statin was associated with a lower risk of sudden cardiac death, but not the risk of ventricular tachyarrhythmias^16^. Nevertheless, all these lines of clinical evidence suggest that dyslipidemia may play an important role in development of ventricular tachyarrhythmias^17^ or sudden cardiac death in patients at risk for or with established cardiovascular diseases.

In experimental studies, copper-oxidized LDL has been shown to modify electrophysiological properties of isolated ventricular myocytes of guinea pigs including prolongation of action potential duration, depolarization of resting membrane potential, spontaneous activity, generation of afterdepolarizations, and modification of transmembrane ion currents^18^. In animal studies, hypercholesterolemia has been consistently shown to be associated with action potential prolongation because of enhanced L-type calcium current (*I*_ca_)^19, 20^. Thus, it was generally believed that both the exogenous copper-oxidized LDL and the endogenous hypercholesterolemia may alter the electrophysiological phenotypes of cardiomyocytes leading to increased or decreased susceptibility to ventricular tachyarrhythmias in the heart^19, 20^. However, the copper-oxidized LDL is not a nature-occurring LDL and the mechanisms underlying the electrophysiological remodeling in cardiomyocytes from hypercholesterolemic animal models are largely speculative.

Recently, we reported that plasma levels of L5, which is endogenous and the most negatively charged subtraction of circulating LDL, are increased in patients with hypercholesterolemia^21^, diabetes^22^, metabolic syndrome^23^, acute myocardial infarction^24, 25^, and stroke^26^. Unlike other subfractions of LDL, L5 is internalized by the lectin-like oxidized LDL receptor-1 (LOX-1) instead of LDL receptor^27^. LOX-1 plays a pathogenic role in myocardial ischemia-reperfusion injury and in atrial tachyarrhythmia^28^. In the present study, we correlated the plasma L5 level with the electrocardiographic QTc variation in patients with coronary artery disease (CAD) and examined the electrophysiological properties of isolated cardiomyocytes from both wild type and LOX-1 knockout mice (LOX-1^−/−^) to delineate the role of LOX-1 involved in the L5-mediated electrical remodeling in cardiomyocytes.

## Results

### Patient characteristics and correlation of L5 with QTc interval

**Figure 1:**
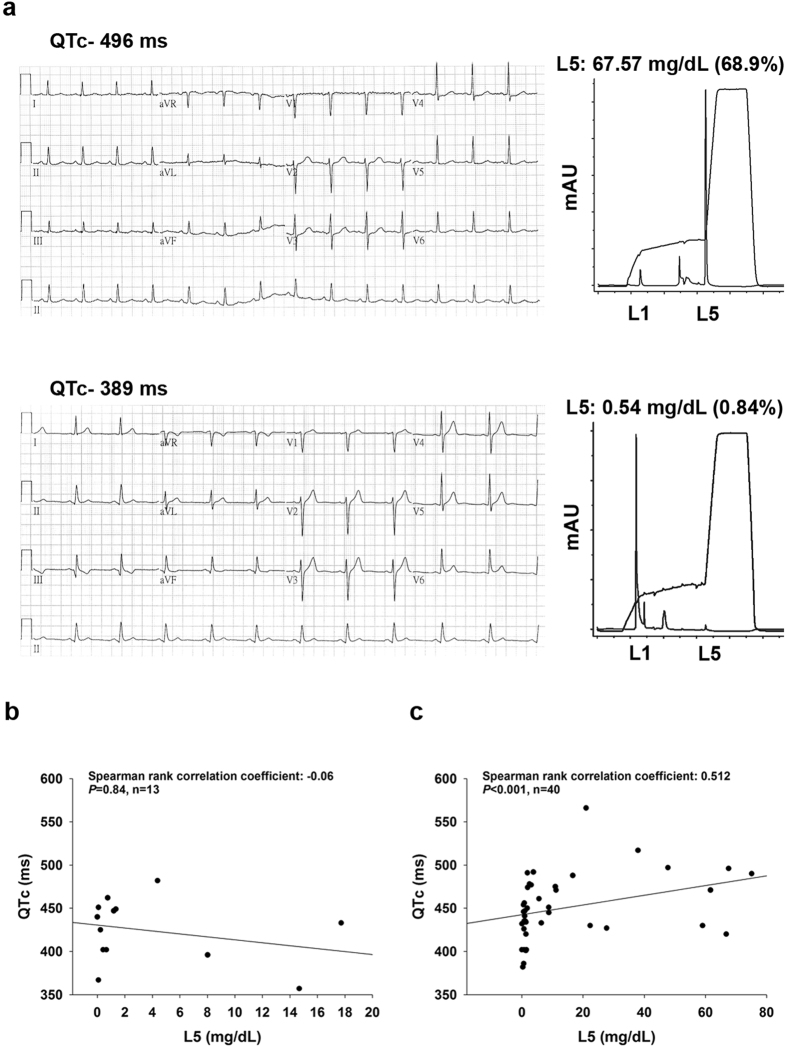
Correlation between L5 and corrected QT interval in humans. (**a**) Representative electrocardiogram shows longer corrected QT interval (QTc) in patient with higher L5 (upper) and shorter QTc in that with lower L5 (lower). (**b**) QTc was plotted against L5 concentration in patients with normal coronary artery (left) and CAD (right).

The demographic and clinical characteristics of the study patients are presented in Table 1. Of the 53 patients, 40 had angiography documented CAD and 13 had no CAD. The median age and the male-to-female ratio were not different between the two groups of patients. The prevalence of established cardiovascular diseases or risk factors such as hypertension, diabetes, hyperlipidemia, cerebral vascular accident, and end-stage renal disease was also comparable between patients with and with no CAD. CAD patients had a higher baseline diastolic blood pressure (140, interquartile range [IQR] 129‒158 vs. 123, IQR 118‒144 mmHg, *P* = 0.04) and a longer QTc (446.50, IQR 429.25‒475.50 vs. 433.00, IQR 402.00‒449.00 ms, *P* = 0.03) compared to controls. The levels of total cholesterol (183.50, IQR 148.25‒199.25 vs. 137.50, IQR 114.75‒152.5 mg/dL, *P* < 0.001), LDL-C (117.55, IQR 77.48‒134.63 vs. 68.00, IQR 57.00‒83.10 mg/dL, *P* < 0.01), and triglyceride (161.50, IQR 117.00‒230.25 vs. 95.00, IQR 70.50‒119.00 mg/dL, *P* < 0.01) were greater and HDL-C was lower (34.30, IQR 30.88‒38.38 vs. 43.95, IQR 41.13‒49.73 mg/dL, *P* < 0.01) in patients with CAD than controls. Notably, the plasma L5 concentration was higher in CAD patients than controls (2.18, IQR 0.89‒17.75 vs. 0.76, IQR 0.23‒4.38 mg/dL, *P* = 0.04). The serum levels of sodium and potassium were normal and equivalent between the two groups of patients. Among the concurrent medications used, only one case (amiodarone prescribed in one patient) was identified to be associated with a known risk of torsades de pointes according to the Arizona Center for Education and Research on Therapeutics (http://www.azcert.org/). With the baseline patient characteristics, we found that the plasma concentration of L5 were positively correlated with QTc in patients with CAD (r = 0.512, *P* < 0.001) but not in patients with normal coronary artery (r = −0.06, *P* = 0.84) (Fig. 1).

**Table 1:** Demographics and Clinical Characteristics

Variables	No CAD n = 13 n (%) or median (IQR)	CAD N = 40 n (%) or median (IQR)	*P* value
Age (years)	55.48 (50.59‒63.81)	59.33 (48.16‒67.31)	0.28
Sex			
Male	6 (46.15)	32 (80)	1.00
Female	7 (53.85)	8 (20)	
History of			
Hypertension	5 (38.46)	26 (65.00)	1.00
Diabetes Mellitus	3 (23.08)	20 (50.00)	0.47
Hyperlipidemia	3 (23.08)	11 (27.50)	1.00
CVD	4 (30.77)	27 (67.50)	0.24
CVA	1 (7.70)	6 (15.00)	1.00
ESRD	2 (15.38)	5 (12.50)	—
Bronchial asthma	0 (0)	3 (6.82)	—
Chronic hepatitis	1 (7.69)	4 (7.50)	—
Gout	0 (0)	1 (2.50)	—
Cancer	2 (15.38)	4 (10.00)	0.32
Smoking	6 (46.15)	28 (70.00)	1.00
BMI (kg/m^2^)	25.93 (21.87‒28.71)	27.64 (24.57‒29.79)	0.21
SBP (mmHg)	123.00 (118.00 ‒144.00)	140.00 (129.00‒158.00)	**0.04**
DBP (mmHg)	80.00 (70.00‒90.00)	81.00 (73.50‒88.00)	0.81
HR (beats/min)	80.00 (69.00‒86.00)	75.50 (64.50‒89.00)	0.25
QT (ms)	358.00 (350.00‒412.00)	409.00 (379.50‒436.00)	0.06
QTc (ms)	433.00 (402.00‒449.00)	446.50 (429.25‒475.50)	**0.03**
TC (mg/dL)	137.50 (114.75‒152.5)	183.50 (148.25‒199.25)	**<0.001**
Triglyceride (mg/dL)	95.00 (70.50‒119.00)	161.50 (117.00‒230.25)	**<0.01**
LDL-C (mg/dL)	68.00 (57.00‒83.10)	117.55 (77.48‒134.63)	**<0.01**
HDL-C (mg/dL)	43.95 (41.13‒49.73)	34.30 (30.88‒38.38)	**<0.01**
L5 (mg/dL)	0.76 (0.23‒4.38)	2.18 (0.89‒17.75)	**0.04**
SGOT (IU/L)	30.00 (23.00‒53.50)	30.00 (18.25‒43.75)	0.38
SGPT (IU/L)	19.00 (16.25‒26.50)	25.00 (14.50‒40.00)	0.60
BUN (mg/dL)^a^	16.00 (14.00‒21.00)	15.00 (12.00‒20.00)	0.42
Creatinine (mg/dL)^a^	0.97 (0.78‒1.00)	0.99 (0.82‒1.23)	0.26
Sodium (meq/L)	137.00 (136.00‒140.00)	137.00 (136.00‒139.00)	0.25
Potassium (meq/L)	3.70 (3.50‒3.70)	3.80 (3.58‒4.20)	0.13
Ischemic heart disease			
STEMI	0 (0)	11 (27.50)	—
Non STEMI	0 (0)	9 (22.50)	—
Unstable angina	0 (0)	4 (10.00)	—
Stable angina/chest pain	13 (100)	16 (40.00)	—
PCI	0 (0)	26 (65.00)	—
Drugs with known TdP risk^a^	0 (0)	1 (2.50)	—
Drugs with possible TdP risk^a^	0 (0)	0 (0.00)	—
Drugs with conditional TdP risk^a^	0 (0)	1 (2.50)	—

CAD, coronary artery disease; IQR, interquartile range; CVD, cardiovascular disease; CVA, cardiovascular accident; ESRD, end-stage renal disease, BMI, body mass index; SBP, systolic blood pressure, DBP, diastolic blood pressure, HR, heart rate; TC, total cholesterol; LDL-C, low-density lipoprotein cholesterol; HDL-C, high-density lipoprotein cholesterol; L5, the most electronegative subfraction of LDL; SGOT, serum glutamic oxaloacetic transaminase; SGPT, serum glutamate pyruvate transaminase; BUN, blood urea nitrogen; STEMI, ST-elevation myocardial infarction; PCI, percutaneous coronary intervention; TdP, torsades de pointes; ^a^according to the Arizona Center for Education and Research on Therapeutics (http://www.azcert.org/).

### L5 prolonged QTc interval via LOX-1 *in vivo*

To determine the direct effect of L5 on QT interval *in vivo*, we intravenously injected wild-type mice with 5 mg/kg L1 or L5 (n = 5 per group) for 1 week and then examined their QTc by performing electrocardiographic analysis. Additionally, LOX-1^−/−^ mice (n = 5) were injected with L5 to determine the role of LOX-1. Table S1 compares the electrocardiographic parameters between the three groups of animals. The QTc were prolonged by about 25% in L5-injected wild-type mice compared to L1 (17.77 ± 0.94 vs. 14.30 ± 1.12 ms; *P* = 0.043), and this L5-mediated QTc-prolonging effect was not seen in L5-injected LOX-1^−/−^ mice. Other electrocardiographic parameters including heart rate, PR interval, RR interval, QRS duration, and QT interval were comparable among the three groups of animals.

### Incubation of L5 prolonged action potential in mice cardiomyocytes

**Figure 2:**
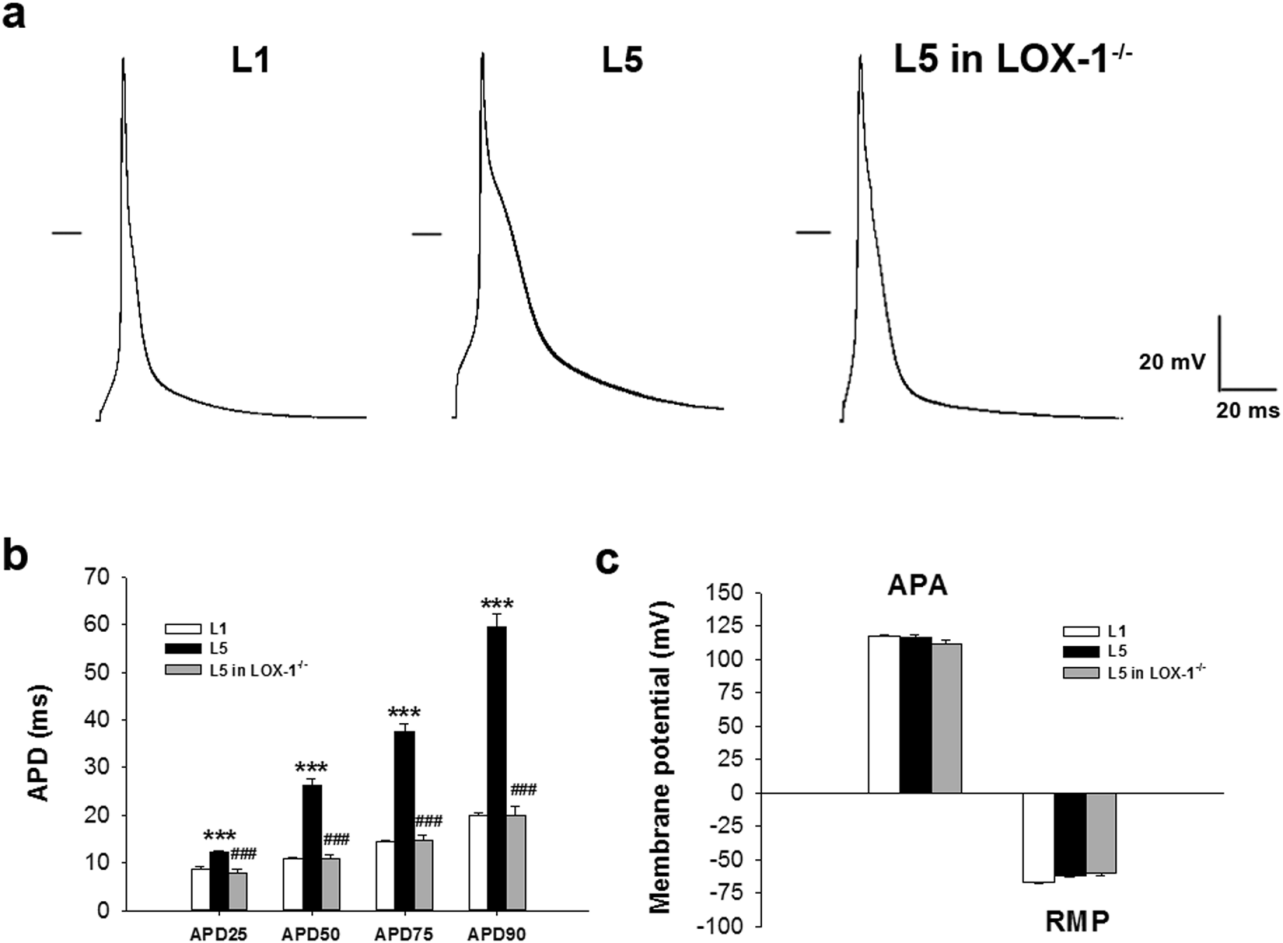
L5 induces prolongation of action potential duration in cardiomyocytes through LOX-1. (**a**) Representative action potentials of 30 μg/mL L1 or L5-treated cardiomyocytes from wild-type mice and L5-treated cardiomyocytes from LOX-1^−/−^ mice. The horizontal line indicates zero voltage level. (**b**) Comparison of the action potential duration at 25% (APD_25_), 50% (APD_50_), 75% (APD_75_), and 90% (APD_90_) repolarization between 3 groups of cardiomyocytes. (**c**) Comparison of the action potential amplitude (APA) and the resting membrane potential (RMP) between 3 groups of cardiomyocytes. n = 6 per group. ***P < 0.01 vs. L1-treated cardiomyocytes; ^###^ *P* < 0.01 vs. L5-treated cardiomyocytes.

To further study the ionic mechanisms involved in QTc prolongation in L5-injected mice, we first compared action potential duration (APD) between 30 µg/mL L1- and L5-treated cardiomyocytes by using the patch-clamp technique. Cardiomyocytes were paced with 3–5 ms suprathreshold depolarizing stimuli in the current-clamp mode. Figure 2a shows the representative recordings of action potentials for wild-type cardiomyocytes treated with L1 or L5 and LOX-1^−/−^ cardiomyocytes treated with L5. The APD at 25%, 50%, 75%, and 90% repolarization was longer in cardiomyocytes incubated with L5 for 30 minutes than that with L1 incubation (*P* < 0.001 for all the 4 comparisons; Fig. 2b). The L5-mediated APD effect was abolished in myocytes isolated from LOX-1^−/−^ mice (*P* < 0.001 for all the 4 comparisons). The action potential amplitude (APA) and resting membrane potential (RMP) were equivalent among three groups of cardiomyocytes (Fig. 2c). Further examination of the relationship between L5 concentration and APD prolongation showed that perfusion with L5 at concentrations of 2.5, 7.5, 25, 75, or 250 µg/mL caused a significant dose-dependent prolongation of APD, with a half-maximal inhibitory concentration (IC_50_) of 54.24 ± 13.2 µg/mL and a Hill coefficient of 0.62 ± 0.009 in H9c2 cells (Fig. S1). The L5-induced prolongation in APD occurred as early as 5 to 10 minutes after the administration of L5 but did not completely recover after 30-min washout. L5 also prolong APD in cardiomyocytes isolated from guniea pig and sheep (Figs S2 and 3), indicating this effect was across species.

### Incubation of L5 increased L-type calcium current (*I*_Ca_) in mice cardiomyocytes

**Figure 3:**
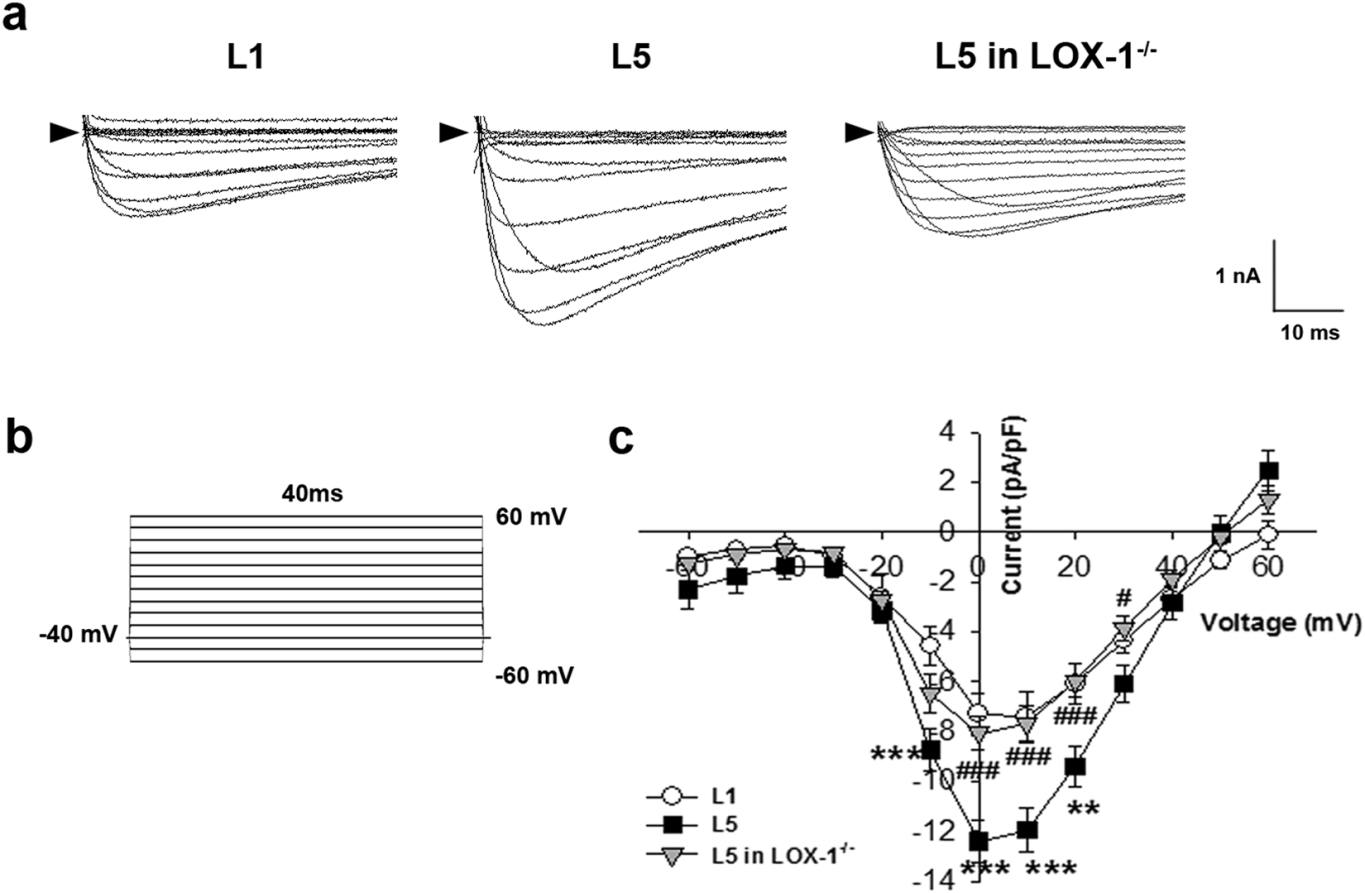
L5 increases L-type calcium current (*I*_Ca_) in cardiomyocytes through LOX-1. (**a**) The original superimposed recordings of inward L-type calcium current in 30 μg/mL L1 or L5-treated cardiomyocytes from wild-type mice and L5-treated cardiomyocytes from LOX-1^−/−^ mice. The arrow in each panel indicates zero current level. (**b**) Schematic diagram of the voltage clamp protocol. (**c**) Comparison of the I-V relationships of *I*_(Ca)_ between 3 groups of cardiomyocytes. n = 6 per group. **P < 0.02 and ***P < 0.01 vs. L1-treated cardiomyocytes; ^#^ *P* < 0.05 and ^###^ *P* < 0.01 vs. L5-treated cardiomyocytes.

The L-type calcium current (*I*_Ca_) of L1 or L5-treated cardiomyocytes were recorded in the voltage-clamp mode (Fig. 3a). Calcium current was elicited by a 40-ms depolarization to various potential levels ranging from −60 to 60 mV (applied at 10 mV increments) from the holding potential of −40 mV (to inactivate sodium channel; Fig. 3b). Current-voltage relationships showed that *I*_Ca_ density was much larger in 30 µg/mL L5-treated cardiomyocytes than in L1-treated cells at −10, 0, 10, and 20 mV (*P* = 0.005, 0.002, 0.006, and 0.018, respectively) after 30 min, and this L5 effect was reversed at 0, 10, 20, and 30 mV in LOX-1^−/−^ cardiomyocytes (*P* = 0.003, 0.004, 0.009, and 0.030, respectively when compared to wild-type cardiomyocytes; Fig. 3c). However, Incubation of L5 with mice cardiomyocytes had no significant effect on the inward sodium current (*I*_Na_) (Fig. S4).

### Incubation of L5 alters I_Ca_ gating properties

**Figure 4:**
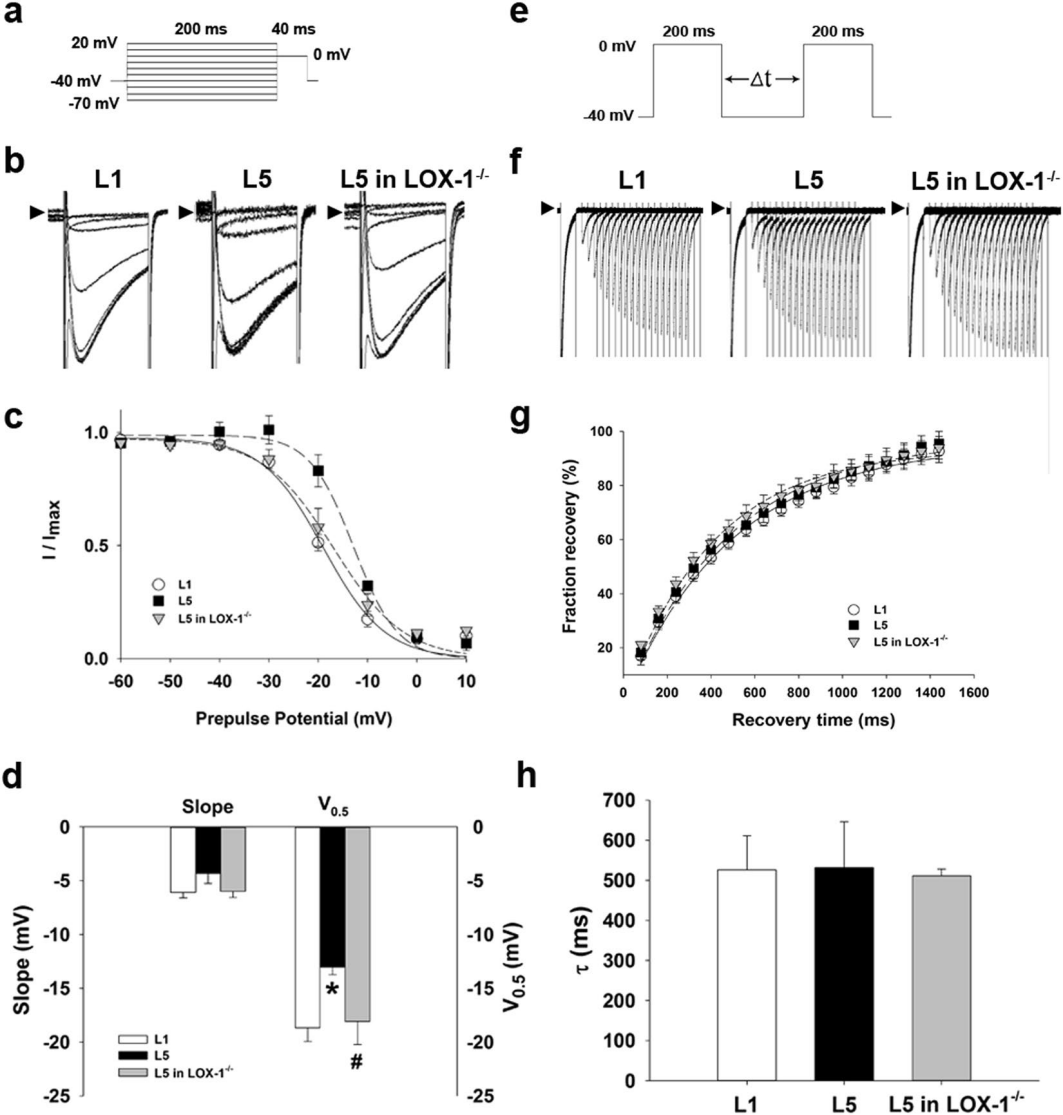
Kinetic change of ICa in L5-treated cardiomyocytes. (**a**) Schematic diagram of the voltage clamp protocol of steady-state voltage-dependence inactivation. (**b**) The original superimposed recordings of steady-state inactivation trace in 30 μg/mL L1 or L5-treated cardiomyocytes from wild-type mice and L5-treated cardiomyocytes from LOX-1^−/−^ mice. The arrow in each panel indicates zero current level. (**c**) The voltage-dependent steady-state inactivation curves for *I*_Ca_ were obtained by normalizing the current amplitudes to the maximal value and plotted as a function of the prepulse (conditioning) potentials. The lines drawn through the data points are the best fit to the Boltzmann equation. (**d**) Histogram comparing slope and midpoint voltage (V_0.5_) of each line between 3 groups of cardiomyocytes. (**e**) Schematic of the typical 2-pulse protocol of kinetics of *I*_Ca_ recovery from inactivation. (**f**) The original superimposed recordings of recovery from inactivation in 3 groups of cardiomyocytes. The arrow in each panel indicates zero current level. (**g**) The recovery from inactivation curves for *I*_Ca_ were obtained and fitted to a single exponential function. (**h**) Histogram comparing the average time constant (τ) for recovery from inactivation between 3 groups of cardiomyocytes. n = 6 per group. *P < 0.05 vs. L1-treated cardiomyocytes; ^#^ *P* < 0.05 vs. L5-treated cardiomyocytes.

We compared the gating properties of *I*_Ca_ between L1- or L5-treated wild-type and L5-treated LOX-1^−/−^ cardiomyocytes to clarify the mechanism underlying *I*_Ca_ upregulation. To examine the voltage dependence of inactivation of *I*_Ca_, we used different voltages of conditioning pulses (−70 to 20 mV), which either hyperpolarized or depolarized from the holding potential of −40 mV for 200 ms to bring the membrane to inactivation; with the second pulse, we depolarized to 0 mV for 40 ms from different precondition pulses (Fig. 4a). Figure 4b shows representative traces of *I*_Ca_ after different preconditioning voltages in 30 µg/mL L1- or L5-treated wild-type and L5-treated LOX-1^−/−^ cardiomyocytes. *I*_Ca_ amplitude normalized to the *I*_Ca_ of the most negative conditioning pulse (*I*_max_) was plotted against conditioning voltage in Fig. 4c, and the curve was fitted by a Boltzmann model to obtain the midpoint of inactivation. We showed that the curve of L5-treated cardiomyocytes was right-shifted and that the midpoint of inactivation was more positive than that of the L1 group (*P* = 0.030). The change of *I*_Ca_ inactivation in L5-treated wild-type cardiomyocytes was reversed in LOX-1^−/−^ cardiomyocytes (*P* = 0.049 when compared to L5-treated wild-type cardiomyocytes; Fig. 4d). For the kinetics of *I*_Ca_ recovery from inactivation, we used a typical 2-pulse protocol. Two identical pulses (from a holding potential of −40 mV to the test potential of 0 mV for 200 ms) were elicited in variable intervals from 80 to 1440 ms in 80-ms increments (Fig. 4e). Representative traces of L1- or L5-treated wild-type and L5-treated LOX-1^−/−^ cardiomyocytes are presented in Fig. 4f, and normalized currents were plotted against the interval between two pulses. The data were fitted by a single exponential equation (Fig. 4g). There were no significant differences in time constant (τ) between L1- and L5-treated wild-type cardiomyocytes and between L5-treated wild-type and LOX-1^−/−^ cardiomyocytes (Fig. 4h).

### Incubation of L5 decreased transient outward potassium current (I_to_) in mice cardiomyocytes

**Figure 5:**
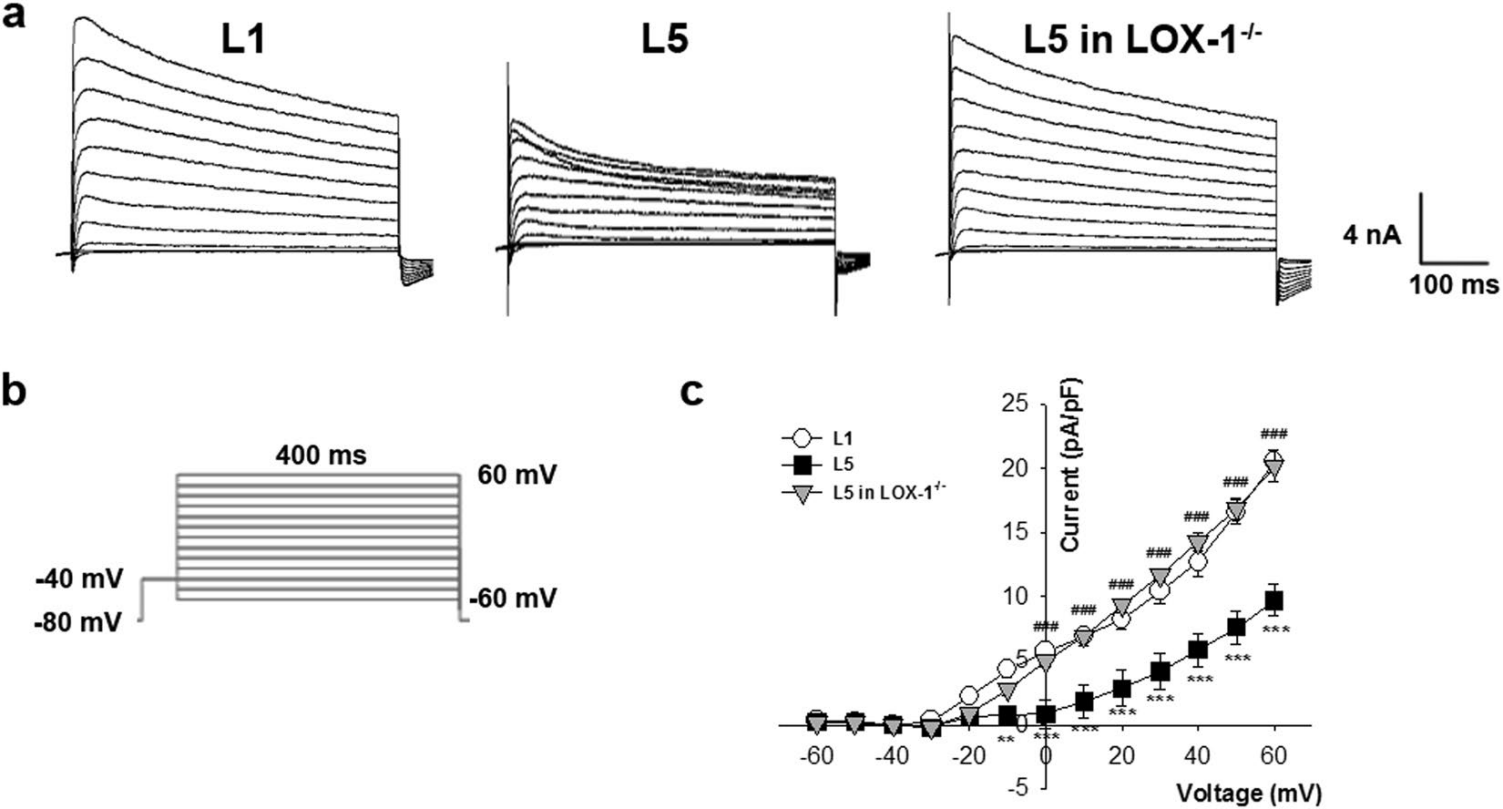
L5 decreases transient outward potassium current (*I*_to_) in cardiomyocytes through LOX-1. (**a**) The original superimposed recordings of outward potassium current in 30 μg/mL L1 or L5-treated cardiomyocytes from wild-type mice and L5-treated cardiomyocytes from LOX-1^−/−^ mice. The arrow in each panel indicates zero current level. (**b**) Schematic diagram of the voltage clamp protocol. (**c**) Comparison of the I-V relationships of *I*_to_ between 3 groups of cardiomyocytes. n = 6 per group. **P < 0.02 and ***P < 0.01 vs. L1-treated cardiomyocytes; ^###^ *P* < 0.01 vs. L5-treated cardiomyocytes.

The transient outward potassium current (*I*_to_) of L1- or L5-treated wild-type and L5-treated LOX-1^−/−^ cardiomyocytes were recorded in the voltage-clamp mode (Fig. 5a). To determine *I*_to_, we elicited cardiomyocytes by a 10-ms step to −40 mV from the holding potential of −80 mV (to inactivate sodium channel), followed by a 400-ms depolarization to various potential levels ranging from −60 to 60 mV (applied at 10 mV increments every 1 s; Fig. 5b). The contamination of the calcium current was prevented by adding 1 mM Co^2+^. *I*_to_ was defined as the difference between the peak value and the current level at the end of a 400 ms pulse. Current-voltage relationships showed that *I*_to_ density was much smaller in 30 µg/mL L5-treated than in L1-treated cardiomyocytes at the potential from −10 to 60 mV (*P* = 0.016 at −10 mV and *P* < 0.001 at 0–60 mV; Fig. 5c) after 30 min, and the L5 effect was not seen in LOX-1^−/−^ cardiomyocytes at the potentials from 0 to 60 mV (*P* < 0.001 compared to L5-treated wild-type cardiomyocytes). Moreover, in cultured H9c2 cell, perfusion with L5 but not L1 significantly decreased the 4-aminopyridine sensitive potassium current (*P* < 0.01), which did not recover immediately after the 30-min washout (Fig. S5). However, Incubation of L5 with mice cardiomyocytes had no significant effect on the inward rectifier potassium current (*I*_K1_) (Fig. S4).

### Incubation of L5 alters I_to_ gating properties

**Figure 6:**
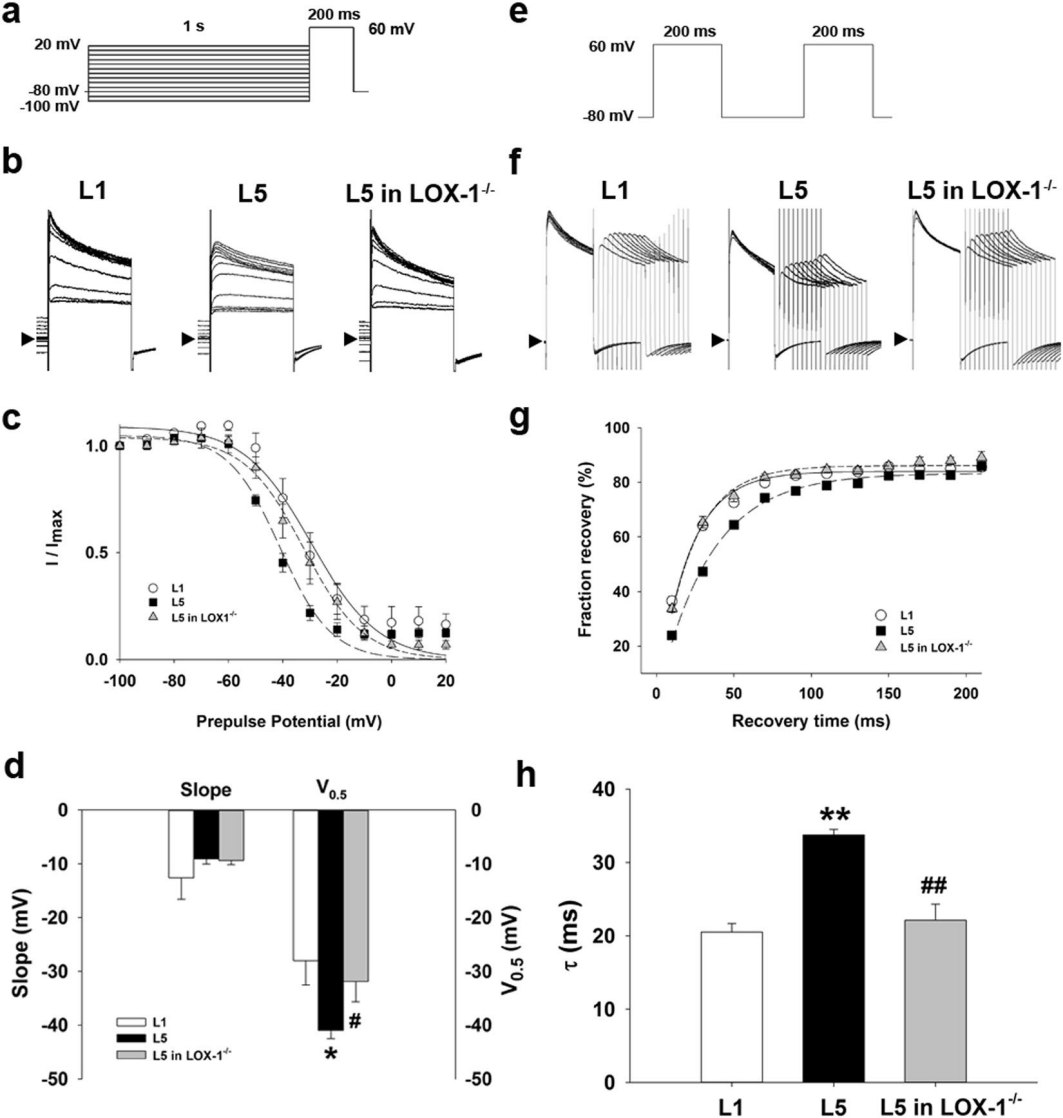
Kinetic change of Ito in L5-treated cardiomyocytes. (**a**) Schematic diagram of the voltage clamp protocol of steady-state voltage-dependence inactivation. (**b**) The original superimposed recordings of steady-state inactivation trace in 30 μg/mL L1 or L5-treated cardiomyocytes from wild-type mice and L5-treated cardiomyocytes from LOX-1^−/−^ mice. The arrow in each panel indicates zero current level. (**c**) The voltage-dependent steady-state inactivation curves for *I*_to_ were obtained by normalizing the current amplitudes to the maximal value and plotted as a function of the prepulse (conditioning) potentials. The lines drawn through the data points are the best fit to the Boltzmann equation. (**d**) Histogram comparing slope and midpoint voltage (V_0.5_) of each line between 3 groups of cardiomyocytes. (**e**) Schematic of the typical 2-pulse protocol of kinetics of *I*_to_ recovery from inactivation. (**f**) The original superimposed recordings of recovery from inactivation in 3 groups of cardiomyocytes. The arrow in each panel indicates zero current level. (**g**) The recovery from inactivation curves for *I*_to_ were obtained and fitted to a single exponential function. (**h**) Histogram comparing the average time constant (τ) for recovery from inactivation between 3 groups of cardiomyocytes. n = 6 per group. *P < 0.05 and **P < 0.02 vs. L1-treated cardiomyocytes; ^#^ *P* < 0.05 and ^##^ *P* < 0.02 vs. L5-treated cardiomyocytes.

We compared the gating properties of *I*_to_ between L1- or L5-treated wild-type and L5-treated LOX-1^−/−^ cardiomyocytes to elucidate the mechanism underlying *I*_to_ downregulation. To examine the voltage dependence of inactivation of *I*_to_, we used different voltages of conditioning pulses (−100 to 20 mV), which either hyperpolarized or depolarized from the holding potential of −80 mV for 1 s to bring the membrane to inactivation; with the second pulse, we depolarized to 60 mV for 200 ms from different precondition pulses (Fig. 6a). Figure 6b shows representative traces of *I*_to_ after different preconditioning voltages in 30 µg/mL L1- or L5-treated wild-type and L5-treated LOX-1^−/−^ cardiomyocytes. *I*_to_ amplitude normalized to the *I*_to_ of the most negative conditioning pulse (*I*_max_) was plotted against conditioning voltage in Fig. 6c, and the curve was fitted by a Boltzmann model to obtain the midpoint of inactivation. We showed that the curve of L5-treated cardiomyocytes was left-shifted and that the midpoint of inactivation was more negative than that of the L1 group (*P* = 0.022). The change of *I*_to_ inactivation in L5-treated wild-type cardiomyocytes was reversed in LOX-1^−/−^ cardiomyocytes (*P* = 0.049 when compared to L5-treated wild-type cardiomyocytes; Fig. 6d). For the kinetics of *I*_to_ recovery from inactivation, we used a typical 2-pulse protocol. Two identical pulses (from a holding potential of −80 mV to the test potential of 60 mV for 200 ms) were elicited in variable intervals from 10 to 210 ms in 20-ms increments (Fig. 6e). Representative traces of L1- or L5-treated wild-type and L5-treated LOX-1^−/−^ cardiomyocytes are presented in Fig. 6f, and normalized currents were plotted against the interval between two pulses. The data were fitted by a single exponential equation (Fig. 6g). It showed that L5 significantly increased time constant (τ) of *I*_to_ fraction recovery in wild-type (33.74 ± 0.77 vs. 20.52 ± 1.15; *P* = 0.016), but not in LOX-1^−/−^ cardiomyocytes (22.13 ± 2.19 vs. 33.74 ± 0.77; *P* = 0.016 compared to L5-treated wild-type cardiomyocytes; Fig. 6h).

### Incubation of L5 decreased hERG current (IKr) in HEK 293 cells

**Figure 7:**
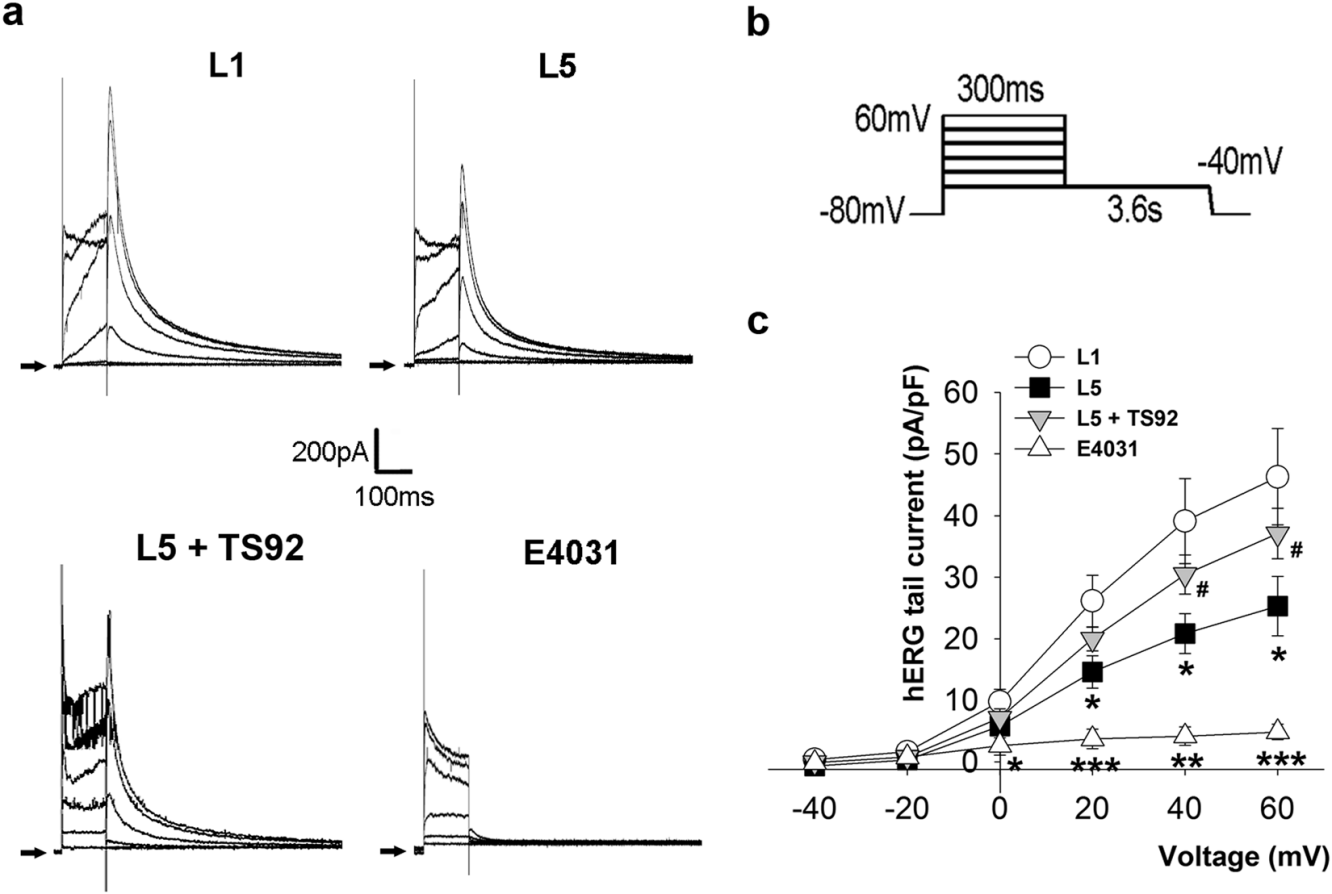
L5 decreases human ether-a-go-go-related gene (hERG) potassium current (IKr) through LOX-1 in HEK293 cells. (**a**) The original superimposed recordings of hERG potassium tail current in 30 μg/mL L1 or L5-treated human embryonic kidney cells transfected with hERG channel. The anti-human monoclonal LOX-1 antibody, TS92 (10 μg/mL) was used to block the LOX-1-dependent effect, and the selective hERG channel blocker E4031 (30 nM) was used as positive control. The arrow in each panel indicates zero current level. (**b**) Schematic diagram of the voltage clamp protocol. (**c**) Comparison of the I-V relationships of *I*_Kr_ between 4 groups of cells. n = 5 per group. *P < 0.05, **P < 0.02, and ***P < 0.01 vs. L1-treated cells; ^#^ *P* < 0.05 vs. L5-treated cells.

To examine the effect of L5 on *I*_Kr_, we used HEK 293 cells transfected with hERG gene encoded *I*_Kr_ channel as the cell model. Figure 7a showed representative traces of hERG tail current in L1 or L5-treated HEK 293 cells. The current was elicited by a voltage protocol shown in Fig. 7b. Incubation of 30 µg/mL L5 for 30 min significantly decreased *I*_Kr_ at the membrane potential of 20, 40, and 60 mV (14.61 ± 2.62 vs. 26.13 ± 4.19, 20.84 ± 3.23 vs. 39.14 ± 6.93, and 25.30 ± 4.84 vs. 46.30 ± 7.78; *P* = 0.048, 0.044, and 0.049, respectively) Pretreatment of anti-human monoclonal LOX-1 antibody, TS92 (10 μg/mL), could abolish the effect of L5. (Fig. 7c). The selective *I*_Kr_ blocker E4031 (30 nM) was used as the positive control to confirm the channel availability.

## Discussion

In the present study, we demonstrate for the first time that the level of the most negatively charged subtraction of circulating LDL, L5, was positively correlated with QTc prolongation in patients with coronary artery disease. Animal studies reveal that exogenous injection of L5 isolated from these patients into wild-type but not LOX-1^−/−^ mice induced QTc prolongation via LOX-1 signaling. Cell studies show that incubation of L5 with cardiomyocytes from mice, sheep, and H9c2 cell line exhibited a universal APD prolongation in a dose-dependent manner. The APD-prolonging effect in mice was caused by LOX-1-mediated enhancement of the sarcolemmal *I*_Ca_ and suppression of *I*_to_. Taken together, L5 can modulate cardiac repolarization via LOX-1-mediated alteration sarcolemmal ionic currents and consequently may play a pivotal role in the arrhythmogenesis in patients with coronary artery disease.

### Cholesterol and cardiac repolarization

Electrocardiographic QT interval represents cardiac repolarization and QT prolongation is an important surrogate marker for increased risk of malignant ventricular arrhythmias and/or sudden death^29^. It has been reported that the sarcolemmal lipid composition can regulate ion channel function and action potential configuration and has important implications for arrhythmogenesis^30, 31^. By using multi-channel magnetocardiography technique to map spatial ventricular repolarization, Huang *et al*. found that QTc dispersion was associated with the total cholesterol levels^32^ in humans. Furthermore, LDL lowering therapy has been associated with a shortening of the QT interval in patients with advanced chronic heart failure, and administration of reconstituted high-density lipoprotein cholesterol leads to a shortening of the QT interval in patients with hypercholesterolemia^33, 34^. Contrarily, QT interval is prolonged in obese patients with high levels of cholesterol^35^. Experimental studies demonstrated that direct exposure of copper-oxidized LDL on cardiomyocytes may lead to APD prolongation and modification of transmembrane ionic currents^36^. In a LDL receptor knockout (LDLr^−/−^) mouse model with elevated serum LDL cholesterol, the QTc interval and the corresponding APD duration were found to be longer in LDLr^−/−^ than in wild-type mice^20^. In present study we found that L5 was positively correlated with QTc prolongation in patients with coronary artery disease. The effect of L5 on the modulation of cardiac repolarization was further confirmed by *in vivo* and *in vitro* studies. The distinct findings in the current study are that a unique LDL subfraction, L5, but not by artificially-oxidized LDL, or by higher levels of total cholesterol/LDL mediate the alteration of the sarcolemmal ionic functions leading to APD prolongation, and LOX-1 signaling may underlie the changes of the cellular electrophysiological properties. All these lines of evidence consistently indicate that cholesterol plays an important role in regulating cardiac repolarization and arrhythmogenosis.

### Electronegative LDL vs. copper-oxidized LDL

Oxidized LDL, which is artificially produced with CuCl_2_, has been shown to cause cardiomyocyte damage and lead to irregular electrical activity in cardiomyocytes^18, 37^. This copper-oxidized LDL has been shown to prolong APD in guinea pig ventricular myocytes and induce afterdepolarization or spontaneous action potentials^18^. Although L5 and oxidized LDL share some chemical similarities, they are fundamentally different in important ways. In contrast to oxidized LDL, which is highly oxidized by an artificial process, L5 is found naturally in patients and is only minimally oxidized^21, 38^. It is noteworthy that the onset of L5’s effects on APD was gradual rather than abrupt. These data are consistent with the time-course analysis of L5’s internalization^21^. Likewise, the effects of L5 did not disappear immediately after a short period of washout (30 min), which can be attributed to the persistence of L5 internalization through LOX-1 with slow degradation of L5. The aforementioned mode of onset and offset is also different that of copper-oxidized LDL-mediated pathway^18^.

### L5 alters repolarization ionic currents to modulate cardiac repolarization

Action potential configuration has been mostly determined by the repolarization current density of inward L-type calcium current and outward transient outward potassium current in the heart^39^. In the present study, we have found that L5 decreased *I*_to_ and increased *I*_Ca_ with resultant lengthening of APD in mice cardiomyocytes. Since *I*_Kr_ plays an important role in repolarization phase of action potential in large mammal cardiomyocytes, it is necessary to examine the effect of L5 on hERG channel. Although the expression of erg mRNA is found in rat hearts, the current amplitude is very small in rodent ventricular myocytes^40^. Thus we used hERG-transfected HEK293 cells to determine the effect of L5 on *I*_Kr_. The results show that L5 reduced *I*_Kr_ in 30 min as expected, which is in parallel to the finding of APD prolongation in sheep cardiomyocytes when incubated with L5, indicating the effect of L5 on the suppression of the repolarizing potassium currents and thus the prolongation of APD is consistent across species. The finding that enhancement of *I*_Ca_ leads to prolongation of APD was concordant with the observation by Zorn-Pauly *et al*.^18^, who showed that oxidized LDL-induced APD prolongation by increasing *I*_Ca_ via oxidative stress-mediated pathway in 6 hours. However, in our present study, both L5-induced APD prolongation and *I*_Ca_ upregulation in cardiomyocytes occur within 30 min of L5 exposure, suggesting that these results are unlikely to be classical oxidative stress-related. Modulation of ion current density could result from altering channel protein expression or changing channel kinetics^41^. In our previous study, we found that incubation of electronegative LDL with rat cardiomyocytes for 24 hours caused *I*_to_ reduction and APD prolongation via downregulation of KChIP2 protein expression. In the current study, we have shown that the there is no significant difference in the membrane fraction of CaV1.2, Kv4.2/4.3, KChIP2, and LOX-1 protein expression between 30 minutes L1- and L5-incubated cardiomyocytes, indicating that the alteration of current density may result from changes of channel kinetics instead of altering protein expression in such a short time (Fig. S6). Indeed, we found that both the voltage-dependent and the time-dependent inactivation kinetics of *I*_Ca_ and *I*_to_ currents were changed in relation to alteration of each current density. This finding further supports the notion that changes of channel kinetics account for the alterations of ionic currents in this study.

### LOX-1-mediated arrhythmogenesis and other possible mechanisms

We showed that knockout of LOX-1 attenuated L5-induced APD prolongation and alterations of sarcolemmal ionic currents, indicating that LOX-1 may mediate the electrophysiological effects of L5 in cardiomyocytes. We previously showed that in a rat model of CKD, the plasma LDL becomes more electronegative which may disrupt SERCA2a-regulated calcium homeostasis and abolish the physiological transmural gradient of *I*_to_ via downregulation of KChIP2 proteins in the epicardial region through LOX-1 signaling pathway^42, 43^. In a rapid atrial pacing pig model, up-regulation of LOX-1 was observed, which was thought to be a response to increased oxidized-LDL formation during pacing-induced oxidative stress^28^. The activation of LOX-1 by oxidized-LDL has been shown to mediate oxidized-LDL-induced abnormal electrophysiological remodeling in ventricular cardiomyocytes^18^. Thus, all these lines of evidence provide a novel insight into the potential role of LOX-1 in directly modulating cardiac electrophysiological properties in addition to its adverse effects on endothelial or myocardial function. A variety of lipid micro-domains, such as caveolae, have been shown to play an important role in protein targeting and modulating protein-protein interactions. Lipid rafts are cholesterol and sphingolipids rich micro-domains of the plasma membrane, and are enriched in signaling molecules and ion channel proteins^44^. Subunits of cardiac channel, such as Kv1.4, Kv1.5, Kv2.1, Kv4, Kir2, Kir3, K_ATP_, Nav and Cav were known to localize in lipid rafts^35^. Since L5 uptake via LOX-1 will shift the balance of intracellular lipids and impacts on membrane protein activity through lipid-dependent process^45, 46^, it is plausible to hypothesize that L5 changes the electrophysiological properties of cardiomyocytes via altering the composition of lipid raft.

### Limitations

Acute myocardial infarction (AMI)/ischemia is known to prolong the QTc interval as well as increase QT dispersion. Therefore, the choice to use the ECGs of patients prior to coronary angiography or coronary intervention may introduce a potential confounder. Further studies are necessary to delineate whether the effect of QTc prolongation is causally related to L5 or is induced by acute myocardial ischemia^47^.

In the current study, we used rodent cardiomyocytes to evaluate the acute electrophysiological effects of L5. However, there are several inherent weaknesses in this model, such as different action potentials morphology and a limited number and type of ion channels compared to humans. Therefore, to confirm the effects of L5 on repolarizing current and APD, we performed additional studies in HEK293 cells transfected with hERG channel and mature sheep cardiomyocytes, respectively. Although the results are consistent, further studies to delineate the underlying ionic mechanisms of APD prolongation by L5 are still needed. Besides, the electrophysiological effect of L5 examined in the present study is within 30 min of incubation with cardiomyocytes, which may not be clinically relevant in humans. Whether chronic incubation of L5 has different effects on the electrophysiological remodeling of cardiomyocytes remain unclear. Lastly, the concentration of L5 used for *in vitro* study was 30 µg/mL, which was equivalent to the clinically relevant range of L5 levels (~30 mg/dL) reported in patients with metabolic syndrome, where L5 composes approximately 25% of total LDL content^42^. However, in the *in vivo* study, the dosage of L5 used to inject mice was 5 mg/kg, which would presumably reach a plasma concentration of 5 mg/dL. Whether injection of a higher dose of L5 would lead to different electrocardiographic phenotype remains to be defined.

In conclusion, we demonstrate that L5 was positively correlated with QTc prolongation in patients with ischemic heart disease. L5 can modulate cardiac repolarization via LOX-1-mediated alteration sarcolemmal ionic currents, and consequently may play a role in the arrhythmogenesis in patients with coronary artery disease.

## Materials and Methods

### Study subjects

Between October 1, 2013 and July 31, 2014, we enrolled 53 consecutive patients with a clinical diagnosis of acute coronary syndrome or angina pectoris who underwent coronary angiography and percutaneous coronary intervention if presence of significant coronary obstruction of ≥70% stenosis. Patients with a Schwartz score^48^ of ≥3.5 were considered having a high probability of congenital long QT syndrome and were excluded from the study^49^. A digital 12-lead ECG was obtained prior to the performance of coronary angiography. When a patient had multiple ECG recordings, the first eligible ECG was used for analysis. All 12-lead ECGs were recorded using a GE Marquette MAC 5500 ECG machine (GE Medical Systems, Milwaukee, WI, USA). The ECGs were inspected by a cardiologist who was blinded to the study to exclude those with technical errors or those that were of inadequate quality before being subjected to QTc analyses. The automated QT interval was measured by the 2001 version of the Marquette 12SL data analysis program (GE Medical Systems, Milwaukee, WI, USA) based on a “Global Median” beat algorithm^50, 51^. The QT interval was then corrected for heart rate using Bazett’s formula. ECGs with wide QRS complex (bundle-branch block, ventricular pacing, or ventricular preexcitation) or significant arrhythmias (atrial fibrillation or bigeminal rhythms) were excluded from the analyses. All participants gave informed consent for the use of their plasma, and the study was approved by the institutional review boards of China Medical University Hospital in Taiwan (DMR 100-IRB-134), and all participants gave written informed consent in accordance with the Declaration of Helsinki.

### LDL isolation and L5 determination in humans

All subjects were instructed to fast before blood sample collection so that lipid concentrations would represent stable lipid levels. Venous blood samples (30 mL) were drawn from each subject by using BD VACUETTE EDTA Blood Tubes (Becton, Dickinson and Company, UK). Human plasma LDL samples were isolated by using sequential potassium bromide density-gradient ultracentrifugation. LDL samples were further resolved into subfractions L1-L5 according to electrical charge and collected by using fast-protein liquid chromatography (GE Health Care, Buckinghamshire, UK) with UnoQ12 anion-exchange columns (BioRad, Inc., Hercules, CA), as described previously^52^.

### *In vivo* animal study

To determine electrical effect of exogenous L5, 8-week-old C57B6/J mice (wild-type mice) were injected with 5 mg/kg of L1 or L5 (n = 5 per group) daily through the tail vein for 1 week. LOX-1 knockout (LOX-1^−/−^) mice (n = 5) from the laboratory of Dr. Tatsuya Sawamura were also injected with L5 daily for 1 week to determine the role of LOX-1. All animal research was approved by the Mackay Medical College Institutional Animal Care and Use Committee (A1020016), and all procedures were conducted in accordance with the Guide for the Care and Use of Laboratory Animals by the US National Institutes of Health. After 1 week, the lead II surface electrocardiogram was recorded in anesthetized animals at the sampling rate of 1000 Hz by using PONEMAH real-time acquisition interface P3P Plus coupled to a digital converter (ML-870, ADInstruments, Colorado Springs, CO, USA). Data were analyzed by the software Lab Chart 7 plus (ADInstruments), and all paramters were measured as the average of five consecutive cycles. The rate-corrected QT interval was calculated according to the Mitchell’s approach verified previously in rodents:

$$\mathrm{ln}\,({\rm{QT}}0)=\,\mathrm{ln}\,({\rm{QTc}})+{\rm{y}}\,\mathrm{ln}\,({\rm{RR}}100)$$

where QT0, QTc, y, RR100 are the observed QT, rate-corrected QT interval, value of the exponent, and normalized RR interval, respectively^53^.

### Isolation of mice cardiomyocytes

Left ventricular cardiomyocytes of mice were enzymatically isolated by the Langendorff perfusion method. Hearts were retrogradely perfused with Krebs buffer (in mM): 120 NaCl, 12 glucose, 25 NaHCO_3_, 1.2 KH_2_PO_4_, 1.2 MgSO_4_, and 5.4 KCl; pH was adjusted to 7.4 by using HEPES. After a 5-min equilibration, 0.4 mg/mL collagenase (type II, Worthington) was added for 20 min. Hearts were then dissected and filtration, the cardiomyocytes were washed twice with Krebs buffer and stored in Krebs buffer. To examine the effect of LDL on cardiomyocytes, we pretreated the cells with LDL for 30 min.

### Transfection of human ether-a-go-go related gene (hERG) in human embryonic kidney (HEK 293) cells

The cloned hERG-pEGFP-N2 vector was a gift from Dr. L.P. Lai’s laboratory. Lipofatamine 2000 (Invitrogen) was used as the reagent for transient transfection. HEK293 cells were seeded into a 35 mm dish and grown in DMEM supplemented with 10% FBS and antibiotics at 37 °C and 5% CO_2_ the day before transfection. Onto the cell monolayer were added 2.5 µg plasmid and 5 µL Lipofatamine 2000 in 1 mL Opti-MEM (Invitrogen). The cells were trypsinized for patch clamp 48 h after transfection.

### Electrophysiological recording

The whole-cell patch-clamp technique was used to record ionic currents and membrane potential with the Axon CNS 700B amplifier (Molecular Devices, LLC, Sunnyvale, CA, USA) with Digidata 1550 A data acquisition system and pClamp software (Version 10, Molecular Devices). A droplet of cell suspension was placed in a chamber mounted on the stage of an inverted microscope (Eclipse Ti-U, Nikon Corporation, Tokyo, Japan) in bath (extracellular) solution containing (in mM) 137 NaCl, 5.4 KCl, 1.8 CaCl_2_, 1.1 MgCl_2_, 6 HEPES, 22 glucose, and 0.33 NaH_2_PO_4_; pH was adjusted to 7.4 using NaOH at room temperature. Only quiescent rod-shaped cells showing clear-cross striations were studied. The mean capacitance of cardiomyocytes was 211.12 ± 6.51 pF. Heat-polished glass electrodes (tip resistances about 1.5 MΩ when filled with pipette internal solution) were prepared from borosilicate glass capillaries (outer diameter 1.5 mm) by the Glass Microelectrode Puller (PC-10, Narishige International Inc., East Meadow, NY, USA). The internal solution contained (in mM) 120 KCl, 5 MgCl_2_, 5 MgATP, 10 HEPES, and 15 EGTA; the pH was adjusted to 7.2 using KOH at room temperature. Junctional potentials were zeroed before the formation of the membrane-pipette seal in the bath solution.

### H9c2 cell culture

H9c2 cells originally derived from embryonic rat heart tissue were obtained from the American Type Culture Collection (ATCC, Manassas, VA) and were cultured in Dulbecco’s modified Eagle’s medium (ATCC) supplemented with 4 mM L-glutamine, 1% nonessential amino acids, 10% fetal bovine serum (ATCC), penicillin (100 IU/mL), and streptomycin (100 µg/mL, Sigma, St. Louis, MO). Cells were cultured in a humidified atmosphere of 5% CO_2_ in air at 37 °C. The concentration of fetal bovine serum was reduced from 10% to 5% during treatments. Patch clamp recording was performed at baseline, during perfusion of L1 or L5, and after 30-min washout with normal Tyrode’s solution. For the acute treatment, cells were perfused with 2.5 to 250 µg/mL of L1 or L5 during patch clamp recording, and normal Tyrode’s solution was used as a control.

### Isolation of cardiomyocytes from sheep and guinea pig hearts

Using a Langendorff system, hearts were perfused with modified calcium-free Tyrode’s solution that contained (in mM): 126 NaCl, 5.4 KCl, 0.8 MgCl_2_, 10 glucose, and 10 HEPES (pH 7.4, adjusted with NaOH). The hearts were then perfused with digestion solution (300 IU/ml collagenase type II and 0.03% protease with 0.1% bovine serum albumin) for 8–10 min, followed by a 10-min washout. The rod-shaped and round myocytes were selected for patch clamp experiments.

### Statistical analysis

The data are expressed as the mean ± standard error of the mean, or median, interquartile range (IQR) as appropriate. The differences between two groups were determined by using the Mann-Whitney U test. Differences between proportions were assessed by chi-square tests or by Fisher’s exact test. A *P*-value < 0.05 was considered statistically significant. The association between L5 concentration and QTc was evaluated by using the Spearman rank correlation coefficient and a stepwise multiple regression model. The inactivation curves of both *I*_Ca_ and *I*_to_ were fitted by using the Boltzmann equation:

$$I/{I}_{{\rm{\max }}}=1/1+\exp [({{\rm{V}}}_{{\rm{m}}}-{{\rm{V}}}_{0.5})/{\rm{k}}]$$

where *I* gives the current amplitude and *I*_max_ its maximum, V_m_ the potential of pulse, V_0.5_ the half-maximal inactivation potential, and k the slope factor. The recovery curves of *I*_Ca_ and *I*_to_ were fitted by the single exponential function:

$${I}_{{\rm{to}}}({\rm{t}})=1-\exp (-{\rm{t}}/{\rm{\tau }})$$

where τ is the time constant of decaying component of inactivation.

## Electronic supplementary material

Supplementary Data

## References

[1] Gordon DJ, Rifkind BM (1989). High-density lipoprotein–the clinical implications of recent studies. The New England journal of medicine.

[2] Castelli WP, Anderson K, Wilson PW, Levy D (1992). Lipids and risk of coronary heart disease. The Framingham Study. Ann Epidemiol.

[3] Verschuren WM (1995). Serum total cholesterol and long-term coronary heart disease mortality in different cultures. Twenty-five-year follow-up of the seven countries study. JAMA: the journal of the American Medical Association.

[4] McNamara DJ (2000). Dietary cholesterol and atherosclerosis. Biochimica et biophysica acta.

[5] de Lemos JA (2004). Early intensive vs a delayed conservative simvastatin strategy in patients with acute coronary syndromes: phase Z of the A to Z trial. JAMA: the journal of the American Medical Association.

[6] Serruys PW (2002). Fluvastatin for prevention of cardiac events following successful first percutaneous coronary intervention: a randomized controlled trial. JAMA: the journal of the American Medical Association.

[7] Stroke Prevetion by Aggressive Reduction in Cholestrol Levels, I., Karam, J. G., Loney-Hutchinson, L. & McFarlane, S. I. High-dose atorvastatin after stroke or transient ischemic attack: The Stroke Prevention by Aggressive Reduction in Cholesterol Levels (SPARCL) Investigators. *J Cardiometab Syndr* **3**, 68–69 (2008). 10.1111/j.1559-4572.2008.07967.x 18326981

[8] Long-Term Intervention with Pravastatin in Ischaemic Disease Study, G. Prevention of cardiovascular events and death with pravastatin in patients with coronary heart disease and a broad range of initial cholesterol levels. *N Engl J Med* **339**, 1349–1357, doi:10.1056/NEJM199811053391902 (1998). 10.1056/NEJM199811053391902 9841303

[9] Randomised trial of cholesterol lowering in 4444 patients with coronary heart disease: the Scandinavian Simvastatin Survival Study (4S). Lancet **344**, 1383–1389 (1994). 7968073

[10] Ridker PM (2008). Rosuvastatin to prevent vascular events in men and women with elevated C-reactive protein. The New England journal of medicine.

[11] Mitchell LB (2003). Are lipid-lowering drugs also antiarrhythmic drugs? An analysis of the Antiarrhythmics versus Implantable Defibrillators (AVID) trial. Journal of the American College of Cardiology.

[12] De Sutter J, Tavernier R, De Buyzere M, Jordaens L, De Backer G (2000). Lipid lowering drugs and recurrences of life-threatening ventricular arrhythmias in high-risk patients. Journal of the American College of Cardiology.

[13] Vyas AK (2006). Reduction in ventricular tachyarrhythmias with statins in the Multicenter Automatic Defibrillator Implantation Trial (MADIT)-II. Journal of the American College of Cardiology.

[14] Goldberger JJ (2006). Effects of statin therapy on arrhythmic events and survival in patients with nonischemic dilated cardiomyopathy. Journal of the American College of Cardiology.

[15] Wanahita N (2012). The effect of statin therapy on ventricular tachyarrhythmias: a meta-analysis. Am J Ther.

[16] Rahimi K, Majoni W, Merhi A, Emberson J (2012). Effect of statins on ventricular tachyarrhythmia, cardiac arrest, and sudden cardiac death: a meta-analysis of published and unpublished evidence from randomized trials. European heart journal.

[17] Liu YB (2006). Dyslipidemia is associated with ventricular tachyarrhythmia in patients with acute ST-segment elevation myocardial infarction. Journal of the Formosan Medical Association = Taiwan yi zhi.

[18] Zorn-Pauly K (2005). Oxidized LDL induces ventricular myocyte damage and abnormal electrical activity–role of lipid hydroperoxides. Cardiovascular research.

[19] Liu YB (2003). Sympathetic nerve sprouting, electrical remodeling, and increased vulnerability to ventricular fibrillation in hypercholesterolemic rabbits. Circulation research.

[20] Baartscheer A (2015). Dyscholesterolemia Protects Against Ischemia-Induced Ventricular Arrhythmias. Circulation. Arrhythmia and electrophysiology.

[21] Chen CH (2003). Low-density lipoprotein in hypercholesterolemic human plasma induces vascular endothelial cell apoptosis by inhibiting fibroblast growth factor 2 transcription. Circulation.

[22] Lu J (2008). Electronegative LDL impairs vascular endothelial cell integrity in diabetes by disrupting fibroblast growth factor 2 (FGF2) autoregulation. Diabetes.

[23] Lee AS (2014). Gender disparity in LDL-induced cardiovascular damage and the protective role of estrogens against electronegative LDL. Cardiovascular diabetology.

[24] Chan HC (2013). Highly electronegative LDL from patients with ST-elevation myocardial infarction triggers platelet activation and aggregation. Blood.

[25] Chang PY (2013). Aspirin protects human coronary artery endothelial cells against atherogenic electronegative LDL via an epigenetic mechanism: a novel cytoprotective role of aspirin in acute myocardial infarction. Cardiovascular research.

[26] Shen MY (2016). Plasma L5 levels are elevated in ischemic stroke patients and enhance platelet aggregation. Blood.

[27] Lu J (2009). Mediation of electronegative low-density lipoprotein signaling by LOX-1: a possible mechanism of endothelial apoptosis. Circulation research.

[28] Goette A (2009). Acute atrial tachyarrhythmia induces angiotensin II type 1 receptor-mediated oxidative stress and microvascular flow abnormalities in the ventricles. European heart journal.

[29] Veglio M, Chinaglia A, Cavallo-Perin P (2004). QT interval, cardiovascular risk factors and risk of death in diabetes. J Endocrinol Invest.

[30] Levitan I, Fang Y, Rosenhouse-Dantsker A, Romanenko V (2010). Cholesterol and ion channels. Subcell Biochem.

[31] Maguy A, Hebert TE, Nattel S (2006). Involvement of lipid rafts and caveolae in cardiac ion channel function. Cardiovascular research.

[32] Chang YC (2015). Early Myocardial Repolarization Heterogeneity Is Detected by Magnetocardiography in Diabetic Patients with Cardiovascular Risk Factors. PloS one.

[33] Vrtovec B (2005). Atorvastatin therapy increases heart rate variability, decreases QT variability, and shortens QTc interval duration in patients with advanced chronic heart failure. Journal of cardiac failure.

[34] Den Ruijter HM (2011). Reconstituted high-density lipoprotein shortens cardiac repolarization. Journal of the American College of Cardiology.

[35] el-Gamal A (1995). Effects of obesity on QT, RR, and QTc intervals. The American journal of cardiology.

[36] Bastiaanse EM, Atsma DE, Kuijpers MM, Van der Laarse A (1994). The effect of sarcolemmal cholesterol content on intracellular calcium ion concentration in cultured cardiomyocytes. Arch Biochem Biophys.

[37] Tousoulis D (2009). Oxidative stress and inflammatory process in patients with atrial fibrillation: the role of left atrium distension. International journal of cardiology.

[38] Yang CY (2003). Isolation, characterization, and functional assessment of oxidatively modified subfractions of circulating low-density lipoproteins. Arteriosclerosis, thrombosis, and vascular biology.

[39] Tomita F, Bassett AL, Myerburg RJ, Kimura S (1994). Diminished transient outward currents in rat hypertrophied ventricular myocytes. Circulation research.

[40] Wymore RS (1997). Tissue and species distribution of mRNA for the IKr-like K^+^ channel, erg. Circulation research.

[41] Yu H (2000). Effects of the renin-angiotensin system on the current I(to) in epicardial and endocardial ventricular myocytes from the canine heart. Circulation research.

[42] Chang KC (2015). Increased LDL electronegativity in chronic kidney disease disrupts calcium homeostasis resulting in cardiac dysfunction. Journal of molecular and cellular cardiology.

[43] Lee AS (2017). Electronegative LDL-mediated cardiac electrical remodeling in a rat model of chronic kidney disease. Sci Rep.

[44] Das M, Das DK (2009). Lipid raft in cardiac health and disease. Current cardiology reviews.

[45] Ke LY, Stancel N, Bair H, Chen CH (2014). The underlying chemistry of electronegative LDL’s atherogenicity. Current atherosclerosis reports.

[46] Ke, L. Y. *et al*. Chemical composition-oriented receptor selectivity of L5, a naturally occurring atherogenic low-density lipoprotein. *Pure Appl Chem* **83**, doi:10.1351/PAC-CON-10-12-07 (2011). 10.1351/PAC-CON-10-12-07 PMC3816395 24198440

[47] Sporton SC, Taggart P, Sutton PM, Walker JM, Hardman SM (1997). Acute ischaemia: a dynamic influence on QT dispersion. Lancet.

[48] Schwartz PJ, Ackerman MJ (2013). The long QT syndrome: a transatlantic clinical approach to diagnosis and therapy. European heart journal.

[49] Crotti L, Celano G, Dagradi F, Schwartz PJ (2008). Congenital long QT syndrome. Orphanet J Rare Dis.

[50] Rautaharju PM, Kooperberg C, Larson JC, LaCroix A (2006). Electrocardiographic predictors of incident congestive heart failure and all-cause mortality in postmenopausal women: the Women’s Health Initiative. Circulation.

[51] Lehtinen AB (2008). Association of NOS1AP genetic variants with QT interval duration in families from the Diabetes Heart Study. Diabetes.

[52] Lee AS (2012). Electronegative low-density lipoprotein induces cardiomyocyte apoptosis indirectly through endothelial cell-released chemokines. Apoptosis: an international journal on programmed cell death.

[53] Mitchell GF, Jeron A, Koren G (1998). Measurement of heart rate and Q-T interval in the conscious mouse. The American journal of physiology.

