# Supplementary material for: Human electronegative low-density lipoprotein modulates cardiac repolarization via LOX-1-mediated alteration of sarcolemmal ion channels
Source: Sci Rep. 2017 Sep 7;7:10889. doi: 10.1038/s41598-017-10503-x (PMC5589822; doi:10.1038/s41598-017-10503-x)
Supplement: Supplementary file 1 — Supplementary Data [file 41598_2017_10503_MOESM1_ESM.pdf]

Human electronegative low-density lipoprotein modulates cardiac repolarization via LOX-1-mediated alteration of sarcolemmal ion channels

An-Sheng Lee, Yutao Xi, Chin-Hu Lai, Wei-Yu Chen, Hsien-Yu Peng, Hua-Chen Chan, Chu-Huang Chen, Kuan-Cheng Chang

Supplementary Figures

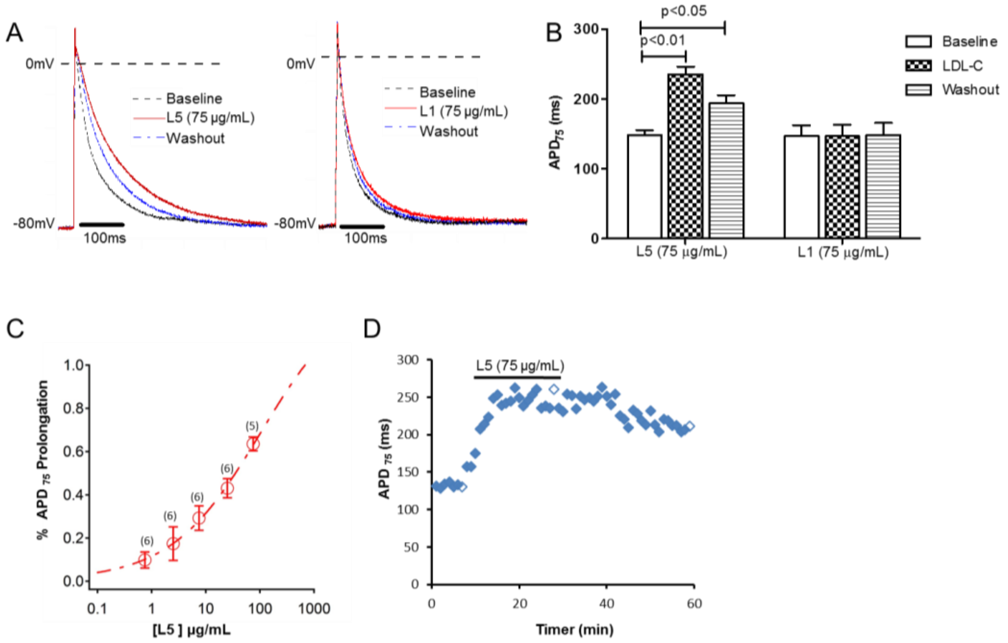

Figure S1. Prolonged action potential duration (APD) in H9c2 cells treated with L5. A, Representative action potential traces of H9c2 cells at baseline, during perfusion with 75 μg/mL L1 or L5, or after 30 min washout. **B,** Perfusion with L5 directly prolonged the APD of H9c2 cells, whereas perfusion with L1 did not. The effects of L5 were only partially reversible. n=8 per group. **C,** L5 acutely prolonged APD in a dose-dependent manner. The prolongation percentage (%) was calculated with respect to the baseline value. When the data were fitted to the Hill Equation, the half-maximal inhibitory concentration (IC50) was 54.24 ± 13.2 μg/mL, and the Hill coefficient was 0.62 ± 0.009. The n number of each concentration was shown in quotes. **D:** Time-course analysis of the effect of L5 (75 μg/mL) on ADP showed that L5 prolonged APD within 5 min.

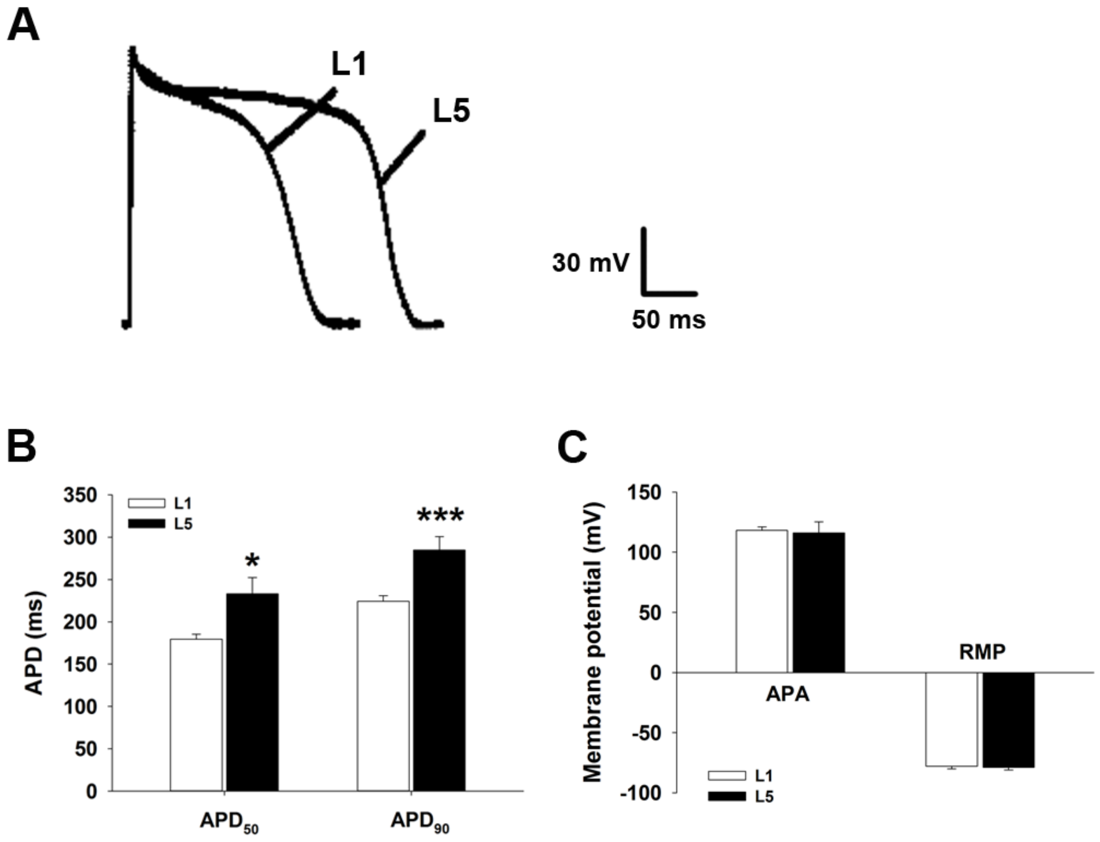

Figure S2. L5-induced prolongation of action potential duration (APD) in ventricular myocytes isolated from guinea pigs. A, Superimposed representative action potential traces were recorded from guinea pig cardiomyocytes treated with 30 μg/mL L5 or L1. **B,** Comparison of the action potential duration at 50% (APD_{50}) and 90% (APD_{90}) repolarization between 2 groups of cardiomyocytes. **C,** Comparison of the action potential amplitude (APA) and the resting membrane potential (RMP) between 2 groups of cardiomyocytes. n=4 per group. *P<0.05 and ***P<0.01 vs. L1-treated cardiomyocytes.

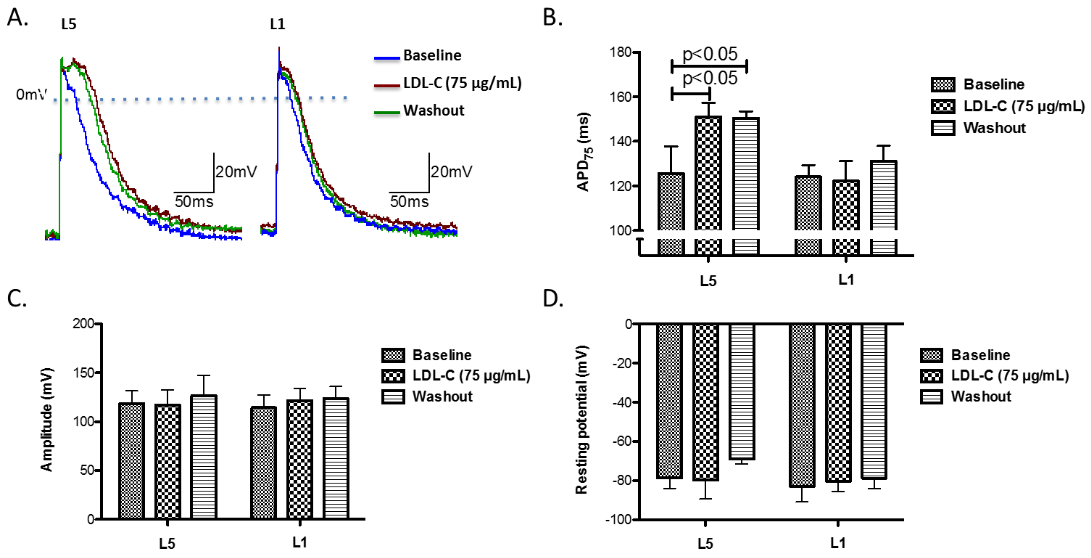

Figure S3. L5-induced prolongation of action potential duration (APD) in myocytes isolated from sheep. A, Representative action potential traces were recorded from sheep myocytes at baseline, during perfusion with 75 μg/mL L5 or L1, and after washout. The direct effects of L5 or L1 perfusion on **(B)** APD, **(C)** amplitude, and **(D)** resting potential in sheep myocytes. n=6 per group.

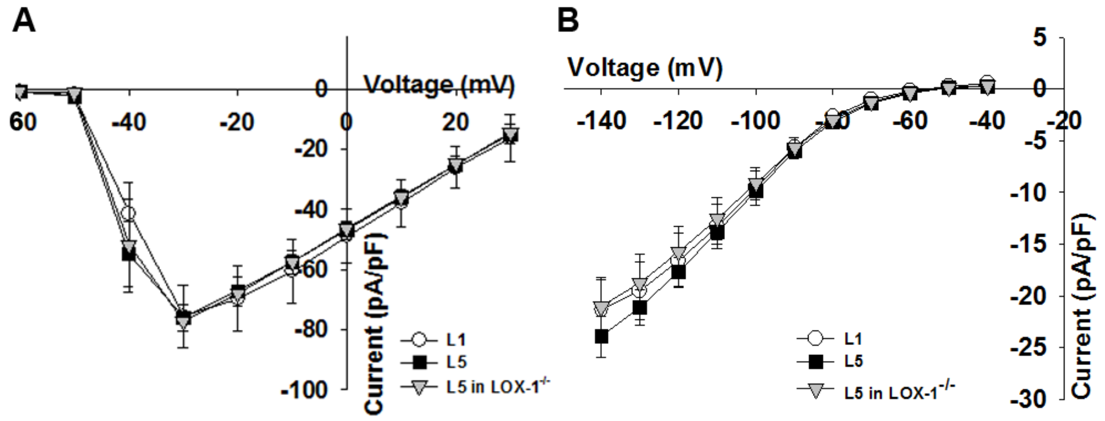

Figure S4. Effect of L5 on sodium (I_{Na}) and inward rectifier potassium current (I_{K1}) in mice cardiomyocytes. A, Comparison of the I-V relationships of I_{Na} between 3 groups of cardiomyocytes. n=3 per group. **B,** Comparison of the I-V relationships of I_{K1} between 3 groups of cardiomyocytes. n=6 per group.

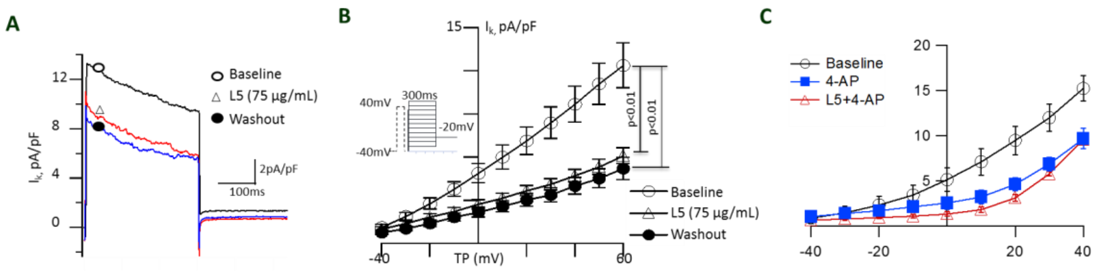

Figure S5. Decreased 4-AP sensitive potassium current (I_K) by L5 in H9c2 cells.

A, Representative outward potassium currents were elicited with voltages ranging from –40 mV to 60 mV (at 10-mV intervals) from a holding potential of –40 mV. **B,** Current-voltage relationship of I_K. **C,** 4-Aminopyridine was used to confirm the potassium current which L5 inhibits is 4-AP sensitive potassium currents. n=7 per group.

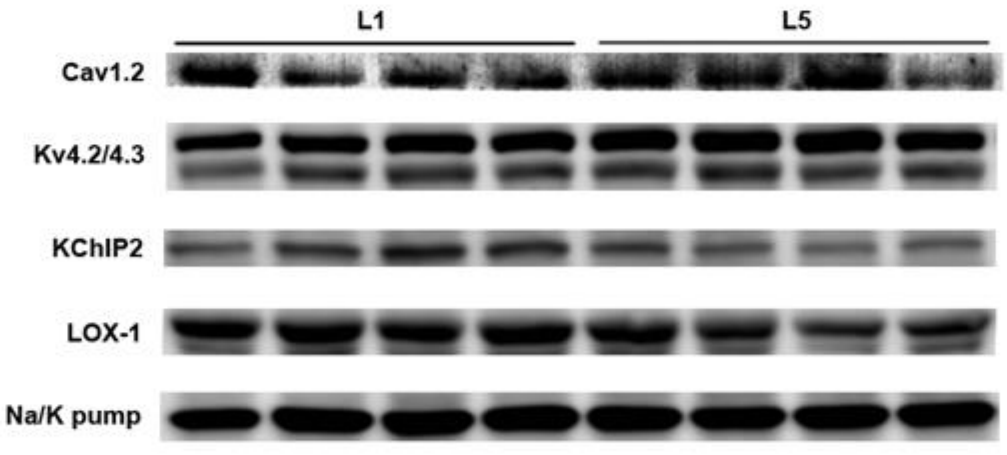

Figure S6. Comparison of membrane fraction proteins between L1- and L5-treated rat ventricular myocytes. It showed that there is no significant difference of Cav1.2, Kv4.2/4.3, KChIP2, and LOX-1 protein expression between 30 min L1- and L5-treated rat ventricular myocytes. Sodium-potassium ATPase (Na/K) was used as membrane fraction internal control.

Supplementary Table

Table S1. Effects of L5 on Electrocardiographic Parameters in Mice

	**L1**	**L5**	**L5 in LOX-1$^{-/-}$**
Heart rate (beats/min)	402.40±52.72	447.44±32.64	409.67±21.24
RR interval (ms)	155.88±17.32	137.15±10.41	147.13±14.60
PR interval (ms)	41.22±3.49	41.34±0.77	40.98±3.33
QRS interval (ms)	10.41±0.84	11.40±0.33	11.22±0.65
QT interval (ms)	17.53±0.54	18.81±1.96	16.61±1.82
QTc (ms)	14.30±1.12	17.77±0.94*	13.41±0.91$^{\#}$

Values are means±S.E. QTc is calculated by the method according to Mitchell GF et al. *P<0.05 vs. L1; $^{\#}P$<0.05 vs. L5.
